# High Mobility Group Box-1 and Pro-inflammatory Cytokines Are Increased in Dogs After Trauma but Do Not Predict Survival

**DOI:** 10.3389/fvets.2018.00179

**Published:** 2018-07-30

**Authors:** Robert Goggs, Jo-Annie Letendre

**Affiliations:** ^1^Department of Clinical Sciences, College of Veterinary Medicine, Cornell University, Ithaca, NY, United States; ^2^Centre DMV, Montreal, QC, Canada

**Keywords:** canine, HMGB-1, IL-6, CXCL8, CCL2, trauma, ATT, APPLE

## Abstract

Trauma is common in dogs and causes significant morbidity and mortality, but it remains challenging to predict the prognosis of dogs with traumatic injuries. This study aimed to quantify plasma high-mobility group box-1 (HMGB-1) and cytokine concentrations in dogs with moderate-to-severe trauma, and to evaluate the association between these biomarkers and the injury severity and survival to discharge. Using a prospective, observational case-control study design, 49 dogs with an animal trauma triage (ATT) score ≥3 were consecutively enrolled from 07/2015 to 10/2017 and followed to hospital discharge. Dogs <3 kg and those with pre-existing coagulopathies were excluded. Thirty three healthy control dogs were also enrolled. Illness and injury severity scores including the acute patient physiologic and laboratory evaluation (APPLE) were calculated using at-presentation data. Plasma HMGB-1 concentrations were measured by ELISA; concentrations of 13 cytokines were measured using multiplex bead-based assays and separately concentrations of 4 cytokines were measured using a multiplex canine-specific ELISA. All biomarkers were measured in duplicate. Mann-Whitney U tests were used to compare biomarker concentrations between groups and between survivors and non-survivors. Associations between biomarkers were evaluated using Spearman's correlation coefficients. Independent predictors of survival were identified using multivariable logistic regression. Alpha was set at 0.05. Plasma concentrations of HMGB-1, interleukin-6, C-X-C motif chemokine-8, keratinocyte chemoattractant-like, and C-C chemokine ligand-2 were significantly greater in injured dogs vs. controls (all *P* ≤ 0.011). In univariate analyses, HMGB-1 was significantly greater in non-survivors 46.67 ng/mL (8.94–84.73) compared to survivors 6.03 ng/mL (3.30–15.75), (*P* = 0.003). Neither HMGB-1 or the cytokines were associated with survival independent of illness severity as measured by the APPLE score, however.

## Introduction

Trauma is a frequent reason for presentation of dogs to veterinary emergency rooms and is a leading cause of morbidity and mortality, particularly in younger dogs (1). Dogs that survive the initial trauma and receive timely intensive care have a better prognosis for survival, while development of complications such as pneumonia, disseminated intravascular coagulation, or multiple organ failure increases the risk of death in these patients (2). Systems such as the Animal Trauma Triage (ATT) score and the canine acute patient physiologic and laboratory evaluation (APPLE) score were developed to aid the initial evaluation of such patients (3, 4). Early and repeated patient scoring can help guide therapeutic interventions and predict survival (5–7), but it remains challenging for clinicians to predict the development of complications or survival in traumatized dogs.

In human trauma patients, biomarkers improve the ability of clinicians to identify occult injury (i.e., those not readily discernible via outward signs or symptoms such as myocardial injury), enable monitoring of response to therapy, and enhance prognostic accuracy (8, 9). Few studies in human or in veterinary medicine have evaluated combinations of biomarkers in trauma. Simultaneous measurement of multiple biomarkers might provide a more detailed picture of the body's reaction to trauma and enhance understanding of the innate immune response to injury (10).

The innate immune system is primed to recognize damage-associated molecular patterns (DAMPs) which are released following tissue trauma (11, 12). These highly conserved molecular motifs initiate inflammatory responses via the Toll-like receptors (TLRs). One such DAMP is high mobility group box-1 (HMGB-1), a DNA-binding nuclear protein and transcription regulator (13), which in the extracellular environment, stimulates the innate immune response via TLRs 2 and 4 (14). Increased concentrations of HMGB-1 are associated with non-survival in human trauma (15). Cells can release HMGB-1 passively such as during necrosis (but not apoptosis) or actively in response to pathogen or cytokine stimulation (13). Passive release occurs rapidly and hence in trauma likely reflects early tissue damage, while the active release occurs more slowly, which makes HMGB-1 a late marker in sepsis. The half-life of HMGB-1 is ~11 h in people (16). Concentrations of HMGB-1 are increased in dogs with gastric dilation-volvulus (GDV) (17, 18), dogs with sepsis (19), and correlate with plasma nucleosome concentrations in critically ill horses (20), suggesting it may provide information about tissue injury in dogs with trauma.

Stimulation of TLRs by DAMPs leads to production of numerous inflammatory mediators including cytokines and chemokines (21). These cytokines include white cell derived interleukins with pro-inflammatory (IL-2, -6, and -18) and anti-inflammatory (IL-4, -10) activities that serve to regulate immune responses, and the chemokines including C-X-C motif chemokine-8 (CXCL8), keratinocyte chemoattractant-like (KC-Like) and C-C chemokine ligand-2 (CCL2) (22) that act to attract leukocytes to sites of infection or tissue damage (23). In human medicine, cytokines have been evaluated as potential trauma biomarkers and have been associated with development of disseminated intravascular coagulation, multiple organ dysfunction syndrome and mortality (24–27). The cytokine profiles induced by lipopolysaccharide (LPS) administration (28, 29), in naturally-occurring SIRS (30), in canine sepsis (19), and in immune-mediated hemolytic anemia (IMHA) (22, 31), have been reported, but to date there are no studies focused on the cytokine profiles of traumatized dogs. Evaluating the cytokine response to trauma could establish patterns of release that may enhance survival prediction (32).

To address these knowledge-gaps and hence improve our understanding of trauma pathophysiology, the present study aimed to quantify the inflammatory state of dogs following trauma through measurement of plasma HMGB-1 and cytokine concentrations. The present study also sought to evaluate the association between illness and injury severity scores and survival to discharge and between plasma cytokine concentrations and survival to discharge in dogs following trauma with the objective of identifying potential prognostic biomarkers. It was hypothesized that dogs following moderate-severe trauma have increased concentrations of HMGB-1 and pro-inflammatory cytokines compared to controls, and that non-survivors have greater concentrations of HMGB-1 and pro-inflammatory cytokines compared to survivors.

## Materials and methods

### Animals

A priori sample size calculations were performed with an online calculator (SISA-Sample Size, Quantitative Skills http://www.quantitativeskills.com/sisa/calculations/samsize.htm), using mortality estimates from published studies (2, 4). The study aimed to identify the difference between the 25th and the 75th percentiles in our pilot data on cell-free DNA concentrations in traumatized dogs. It was estimated that 42 dogs at 14% mortality would enable detection of a significant difference between these percentiles. It was intended that an additional 6 dogs (15%) be enrolled to allow for withdrawal or loss to follow-up. Sample size calculations for HMGB-1 or cytokine concentrations, were not performed, because pilot data were not available. Blood samples were collected at hospital admission from 49 dogs following moderate-to-severe trauma (ATT ≥3) enrolled between 07/2015 and 10/2017. The ATT has been validated for outcome prediction in dogs following trauma (33), and was the sole scoring system used to identify potential study candidates. Details of all scoring systems used in the present study are presented as supplementary material Data Sheet 1. Additional details on other biomarkers measured in samples from the dogs described in this study are presented in an accompanying manuscript (34).

Dogs weighing <3 kg and those with a known, pre-existing coagulopathy were excluded to minimize risks associated with blood sampling. Respective attending clinicians determined all aspects of patient management but were not made aware of patient biomarker concentrations. All patient management decisions were individualized and no care was protocolized; however, all care was provided under the supervision of board-certified specialists or under their direction by residents in training. Apart from blood samples collected as part of the present study, all other diagnostic testing was at the discretion of the attending clinician. For the injured dog population, the primary outcome measure was death or euthanasia for disease severity prior to hospital discharge. Dogs euthanized for financial limitations were excluded from survival analyses. Study participation was undertaken with written informed client consent with local IACUC approval (2014-0053). A population of healthy dogs (*n* = 33) was also recruited, also with written informed consent under local IACUC approval (2014-0052). Healthy controls dogs were eligible if they had no history or evidence of recent or chronic medical conditions and had not received any medication, except for routine preventative healthcare, within the preceding 3 months. Dogs were classified healthy on the basis of history, physical examinations, and complete blood count and serum chemistry results.

### Data collection

Signalment, previous medical history and physical examination findings at hospital admission were recorded. At admission, non-invasive blood pressure measurement by oscillometric (Cardell 9401, Midmark, Dayton, OH) or Doppler methods and pulse oximetry (Rad-87, Masimo, Irvine, CA) were performed. Mentation, modified Glasgow coma scale (MCGS) (35) and ultrasound body cavity fluid scores were measured to enable calculation of the APPLE and APPLE_fast_ scores (6). Where missing data needed for illness severity scoring were encountered, the median value for the remainder of the population was used instead. The shock index, sequential organ failure assessment (SOFA) and survival prediction index-2 (SPI2) scores were calculated as previously reported (5, 7, 36). Blood samples were collected at enrollment for blood lactate (Lactate Pro, Arkray, Edina, MN), complete blood count (ADVIA 2120, Siemens, Washington, DC), serum biochemistry profiles (Modular P, Roche-Hitachi, Indianapolis, IN), and for measurement of HMGB-1 and cytokine concentrations. The maximum quantity of additional blood collected for this study was 5.4 mL (2 × 2.7 mL citrate tubes). For the HMGB-1 and cytokine measurements, citrated plasma was generated by centrifugation of whole blood for 10 min at 1,370 g (Ultra-8V Centrifuge, LW Scientific, Lawrenceville, GA). After centrifugation, plasma was decanted into polypropylene freezer tubes (Polypropylene Screw-Cap Microcentrifuge Tubes, VWR, Radnor, PA), by pipetting. Some plasma was deliberately left in the tube to minimize the risk of disturbing the buffy coat. Residual plasma was then immediately frozen (−20°C) overnight before being transferred to long-term storage (−80°C) until cytokine analyses were performed.

Concentrations of plasma HMGB-1 were analyzed using a commercial ELISA (HMGB-1 ELISA, Tecan US, Morrisville, NC), as previously reported (17). Per the manufacturer, this assay can be affected by hemolysis, but only 4 dogs in the present study had evidence of hemolysis (hemoglobin >100 mg/dL [1 g/L]). An antibody-coated microsphere-based multiplex cytokine immunoassay kit (CCYTOMAG-90K, EMD-Millipore, Billerica, MA) designed for the simultaneous quantification of 13 cytokines was used to measure cytokines as previously described (31). Analytes included in the kit were IL-2, IL-6, IL-7, IL-10, IL-15, IL-18, TNF-α, interferon-γ (IFN-γ), granulocyte macrophage-colony stimulating factor (GM-CSF), KC-Like, CCL2, CXCL8, and CXCL10. Cytokine concentrations were measured in duplicate and the mean values used for subsequent analyses. The observed concentration of each analyte for each sample was calculated using a standard curve generated from the standards and blank provided by the manufacturer [the median (min-max) *r*^2^ values for standard curves was 1.000 (0.996–1.000)]. Sample concentrations that were extrapolated outside standard curve values were used as the calculated concentrations. The manufacturer stated minimum detectable concentrations in pg/mL were: 21.0 (CCL2), 21.7 (CXCL8), 3.2 (CXCL10), 9.2 (GM-CSF), 13.6 (IFN-γ) 3.5 (IL-2), 3.7 (IL-6), 7.5 (IL-7), 8.5 (IL-10), 9.0 (IL-15), 5.8 (IL-18), 5.3 (KC-like), 6.1 (TNF-α).

To enhance sensitivity, concentrations of four cytokines IL-2, IL-6, CXCL8 and TNF-α were also measured using a second, distinct multiplex assay method as previously reported (37, 38). These four cytokines were measured simultaneously using a canine-specific multiplex ELISA (Canine ProInflammatory Panel 3 Ultra-Sensitive Kit, Meso Scale Diagnostics, Rockville, MD) conducted per the manufacturer's instructions. Detection of the cytokines was achieved using electrochemiluminescence using a proprietary reader (QuickPlex SQ 120, Meso Scale Diagnostics, Rockville, MD). The concentrations of each cytokine were measured in duplicate and the mean values used for subsequent analyses.

### Statistical methods

Prior to test selection, data were assessed for normality by assessment of histograms, calculation of skewness and kurtosis and with the D'Agostino Pearson test and descriptive statistics calculated. The majority of variables were not normally distributed and hence all are reported as median (interquartile range). Continuous variables were compared between groups (e.g., controls vs. trauma cases, survivors vs. non-survivors) with the Mann-Whitney U test. Associations within and between biomarker concentrations and vital signs, illness severity scores and patient parameters were evaluated with scatterplots and by calculation of Spearman's correlation coefficients (r_s_). Strength of correlation was assessed as follows: 0.0–0.2 slight, 0.2–0.4 mild, 0.4–0.6 moderate, 0.6–0.8 moderately strong, 0.8–1.0 strong. For categorical variables, 2 × 2 contingency tables were constructed to compare frequencies and were analyzed by Fisher's exact test and the calculation of odds ratios (OR). Predictors of non-survival at hospital discharge were evaluated by plotting receiver-operating characteristic (ROC) curves and through calculation of the area-under these curves (AUROC). Multivariable logistic regression was used to determine if concentrations of HMGB-1 or the cytokines predicted survival to discharge independent of disease severity based on the APPLE score. Candidate predictor variables identified with univariate analyses were entered using a forward stepwise method, using *P* < 0.05 for the likelihood ratio to add explanatory variables to the model. Model accuracy was determined using 2 × 2 classification tables. Model discrimination was determined by calculating AUROC. Model calibration was assessed by Hosmer–Lemeshow goodness-of-fit (model rejected if *P* < 0.05) and visual inspection of contingency tables. Model utility was assessed using Nagelkerke's *R*^2^. The optimal cut off for sensitivity and specificity was identified by maximizing the Youden index (J) where J = (Sensitivity + Specificity)−1 (39). All analyses were performed using commercial software (Prism 6.0, GraphPad, La Jolla, CA; SPSS Statistics 24, IBM, Armonk, NY). Alpha was set at 0.05.

## Results

### Demographics

The study enrolled 49 dogs following trauma, and 33 healthy control dogs. Among the injured dogs, there were 16 mixed breed dogs, 6 Labrador retrievers, 3 German shepherd dogs, 2 border collies, 2 German short-haired pointers, 2 Rhodesian ridgebacks, and 2 Staffordshire bull terriers. There were 16 other pure breed dogs, all *n* = 1. There were 16 male neutered dogs, 9 male intact dogs, 16 female spayed dogs and 8 female intact dogs. The median age was 4 years (1.15–7.75) and the median bodyweight was 24.0 kg (10.0–35.0). The healthy dogs consisted of 18 mixed breed dogs, 4 Labrador retrievers, 2 German short-haired pointers, 2 Golden retrievers, 2 Staffordshire bull terriers, and 4 other pure breed dogs. There were 12 castrated male dogs, 5 male intact dogs, 15 female spayed dogs, and 1 female intact dog. The median age was 3 years (1.95–5.25) and the median bodyweight was 23.6 kg (15.1–31.2). Age, weight, and proportion of males vs. females was not significantly different between the trauma group and the controls.

In the trauma dog population, the estimated median time interval between trauma and presentation was 2.5 h (1–5). There was a significantly longer time interval between injury and study enrollment for patients that were referred than for walk-in cases [4.75 h (3–15) vs. 1 h (0.7–2), *P* < 0.0001]. There was no significant difference in ATT scores (*P* = 0.794) or in APPLE scores (*P* = 0.747) between walk-in cases and referrals. The mechanism of trauma was blunt force impact (hit by car) in 81.6% (40/49), dog bites in 14.2% (7/49), crush injury (*n* = 1), and fall from moving vehicle (*n* = 1). Following injury, 46.9% (23/49) were assessed by a primary care veterinarian before being referred to the study institution. The other 26 dogs (53.1%) presented directly to the study hospital. Of the 23 dogs seen by a primary care veterinarian prior to referral, 18 dogs received 43 treatments in total, median 2 (1–3). Treatments included opioid analgesia (*n* = 11), fluid therapy (*n* = 9), antimicrobials (*n* = 6), glucocorticoids (*n* = 5), hyperosmolar agents (*n* = 3), unspecified analgesics (*n* = 3), alpha-2-agonists (*n* = 2), non-steroidal anti-inflammatory drugs (*n* = 2), antiemetics (*n* = 1), gastroprotectants (*n* = 1). Radiographs were performed in 4 dogs prior to referral. Patient vital parameters, injury severity scores and the results of initial point-of-care assessments recorded at presentation to the study institution are summarized in Table 1. Of these parameters, only temperature was significantly different between dogs that were referred 100.3°F (98.8–101.1) [37.9°C (37.1–38.4)] compared to those that presented primarily to the study institution 101.3°F (100.5–101.8) [38.5°C (38.1–38.8)] (*P* = 0.0095). There were 3 dogs that did not have a complete blood count or a serum chemistry panel available. For these patients, the median values for albumin, creatinine, and total bilirubin (*n* = 46) were used to enable APPLE score calculation and the median platelet count (*n* = 46) was used to enable APPLE_fast_ score calculation. There were 3 dogs where the only blood pressure reading available was obtained by Doppler methodology. For these 3 patients these values were used for SPI2 score calculation. Results of complete blood counts and serum chemistry panels are summarized in Table 2 (for SI units, see Supplementary Table 1).

**Table 1:** Summary statistics for patient vital parameters, injury severity scores and the results of initial point-of-care assessments recorded at presentation to the study institution.

**Parameter**	***n***	**Mean**	***SD***	**Min**	**25th%**	**Median**	**75th%**	**Max**
T (°F)^*^	49	100.6	1.4	97.5	99.7	100.9	101.5	103
T (°C)	49	36.9	6	8.2	37.5	38.2	38.6	39.4
PR (bpm)^*^	49	138	38	60	111	140	163	220
RR (rpm)	49	42	21	12	28	36	51	100
SpO_2_ (%)	48	95	5	74	94	96	98	100
SAP (mmHg)	49	139	33	78	116.5	139	159.5	250
MAP (mmHg)^†^	46	103	24	61	86	100	118	177
Lactate (mmol/L)	49	3	1.8	0	1.7	2.7	3.6	7.9
Shock index	49	1.07	0.46	0.34	0.78	1.02	1.35	2.58
Fluid score	49	NA	NA	0	0	0	1	1
MGCS	49	NA	NA	7	15	16	17	18
Mentation	49	NA	NA	0	1	2	3	4
ATT	49	NA	NA	3	4	5	6	13
SPI2	49	NA	NA	0.66	1.65	2.19	2.63	3.67
APPLE	49	NA	NA	6	20	26	33	49
APPLE_fast_	49	NA	NA	12	19	23	28	40
SOFA	49	NA	NA	0	0.5	1	2	6

* These parameters were normally distributed; the remainder were non-parametric.

† *For the MAP, only 46 values were available because 3 dogs had only a Doppler blood pressure recorded. Doppler blood pressure readings were included in the SAP statistics. APPLE, acute patient physiologic and laboratory evaluation; ATT, acute trauma triage score; MAP, mean arterial pressure; MGCS, modified Glasgow coma scale score; PR, pulse rate; RR, respiratory rate; SAP; systolic arterial pressure; SOFA, sequential organ failure assessment score; SPI2, survival prediction index-2; SpO_2_, oxygen saturation; T, temperature*.

**Table 2:** Summary statistics for complete blood count and serum chemistry panels collected at presentation to the study institution.

**Parameter**	***n***	**Min**	**25%**	**Median**	**75%**	**Max**	**Mean**	**SD**
Hematocrit (%)^*^ (41–58)	46	27	40	47	53.5	72	46.13	9.4
Leukocytes (× 10^3^/μL) (5.7–14.2)	46	6.4	9.175	10.95	14.9	26.4	12.96	5.6
Neutrophils (× 10^3^/μL) (2.7–9.4)	46	3	6.5	8.7	13.25	22.9	10.57	5.6
Band neutrophils (× 10^3^/μL) (0.0–0.1)	46	0	0	0	0.3	1.2	0.23	0.4
Lymphocytes (× 10^3^/μL) (0.9–4.7)	46	0.3	0.8	1.2	1.825	3.5	1.36	0.8
Monocytes (× 10^3^/μL) (0.1–1.3)	46	0.1	0.275	0.5	0.825	2.3	0.65	0.6
Platelets (× 10^3^/μL)^*^ (186–545)	46	60	181	239	300.3	487	245	96
Sodium (mmol/L)^*^ (143–150)	46	143	147	148	151	157	149	3.2
Potassium (mmol/L) (4.1–5.4)	46	3.2	3.875	4.1	4.225	5.2	4.07	0.4
Chloride (mmol/L) (106–114)	46	102	107.8	109.5	114	127	110.8	5.0
Bicarbonate (mmol/L) (14–24)	46	7	17	19.5	22	25	19.07	3.5
Anion Gap (mmol/L) (17–27)	46	15	20	23.5	26	40	23.15	4.5
BUN (mg/dL) (9–26)	46	4	14.25	19	23	44	18.92	7.1
Creatinine (mg/dL) (0.6–1.4)	46	0.3	0.6	0.8	1	2	0.87	0.4
Calcium (mg/dL)^*^ (9.4–11.1)	46	7.5	9.15	9.75	10.5	11.3	9.7	1.0
Phosphate (mg/dL)^*^ (2.7–5.4)	46	1.5	3.2	4.35	5.35	9.3	4.56	1.9
Magnesium (mEq/L) (1.5–2.1)	46	1.2	1.5	1.7	1.9	3.1	1.75	0.4
Total Protein (g/dL)^*^ (5.5–7.2)	46	2.3	4.725	5.4	6.4	9.4	5.48	1.3
Albumin (g/dL)^*^ (3.2–4.1)	46	1.5	2.875	3.3	3.525	4.4	3.19	0.6
Globulin (g/dL)^*^ (1.9–3.7)	46	0.8	1.675	1.95	2.7	3.8	2.16	0.7
Glucose (mg/dL) (68–104)	46	85	105	117.5	151.5	295	134.2	43.3
ALT (U/L) (17–95)	46	14	98.75	364.5	1129	6273	899.8	1333
AST (U/L) (18–56)	46	47	125.3	338	1318	4306	902.3	1135
ALP (U/L) (7–115)	46	17	41.75	67	136.5	834	113.9	137.1
GGT (U/L) (0–8)	46	3	3	3	6.25	18	5.3	3.7
T. Bilirubin mg/dL)^*^ (0.0–0.2)	46	0	0	0.1	0.2	0.4	0.12	0.1
Cholesterol (mg/dL)^*^ (136–392)	46	63	152.5	201	257.5	351	197.4	69.2
CK (U/L) (64–314)	46	301	1,398	3,371	7,050	51,217	6,765	9,790

* *These parameters were normally distributed; the remainder were non-parametric. ALT, alanine aminotransferase; ALP, alkaline phosphatase; AST, aspartate aminotransferase; CK, creatine kinase; GGT, gamma-glutamyl transpeptidase; T. Bilirubin, total bilirubin. Institution reference intervals are indicated in parentheses*.

### Patterns of injury

Within the 49 dogs enrolled following trauma, 164 separate injuries were described. Skin wounds (including bite wounds, lacerations, degloving injuries, and abrasions) were the most frequently reported injury (*n* = 25). Fractures were frequently reported, affecting the appendicular skeleton (*n* = 19), pelvis (*n* = 18), spine (*n* = 8), facial bones (*n* = 7), dentition (*n* = 5), ribs (*n* = 4), hard palate (*n* = 1), hyoid apparatus (*n* = 1), cranial vault (*n* = 1), and scapula (*n* = 1), while luxations of the sacroiliac (*n* = 6), coxofemoral (*n* = 5), elbow (*n* = 1), and glenohumeral (*n* = 1) joints were also common. Other injuries included hemoabdomen (*n* = 13), pneumothorax (*n* = 12), pulmonary contusions (*n* = 11), surgically confirmed abdominal visceral injury (*n* = 4), traumatic brain injury (*n* = 4), diaphragmatic rupture/hernia (*n* = 2), hemothorax (*n* = 2), pneumomediastinum (*n* = 2), retroperitoneal hemorrhage (*n* = 2), arrhythmia (*n* = 1), brachial plexus avulsion (*n* = 1), collateral ligament rupture (*n* = 1), and globe rupture (*n* = 1).

### Survival

The mortality rate for the injured dogs was 20.4% (10/49) of which 9 (90%) were euthanized and 1 (10%) died naturally. No dogs were euthanized for financial limitations; all dogs were euthanized due to injury severity. Of the 10 dogs that were euthanized, 7 were euthanized prior to hospitalization (i.e., within hours of presentation). All 7 of these dogs suffered polytrauma including multiple fractures, brain, or spinal cord injury and concurrent soft tissue injury such as hemoabdomen, hemothorax or ureteral avulsion. Among the 3 hospitalized non-survivors, 2 dogs were euthanized and 1 dog died; none of these dogs survived more than 1 day. Among the survivors the median duration of hospitalization was 4 days (2–5) prior to discharge. Two dogs were discharged against medical advice (these dogs were excluded from the duration of hospitalization calculations). A significantly greater proportion of dogs that presented primarily to the study institution died or were euthanized (34.6 %), compared to those that were referred (4.3 %) (OR 11.65, 95% CI 1.76–132.70, *P* = 0.012).

### HMGB-1

**Figure 1:**
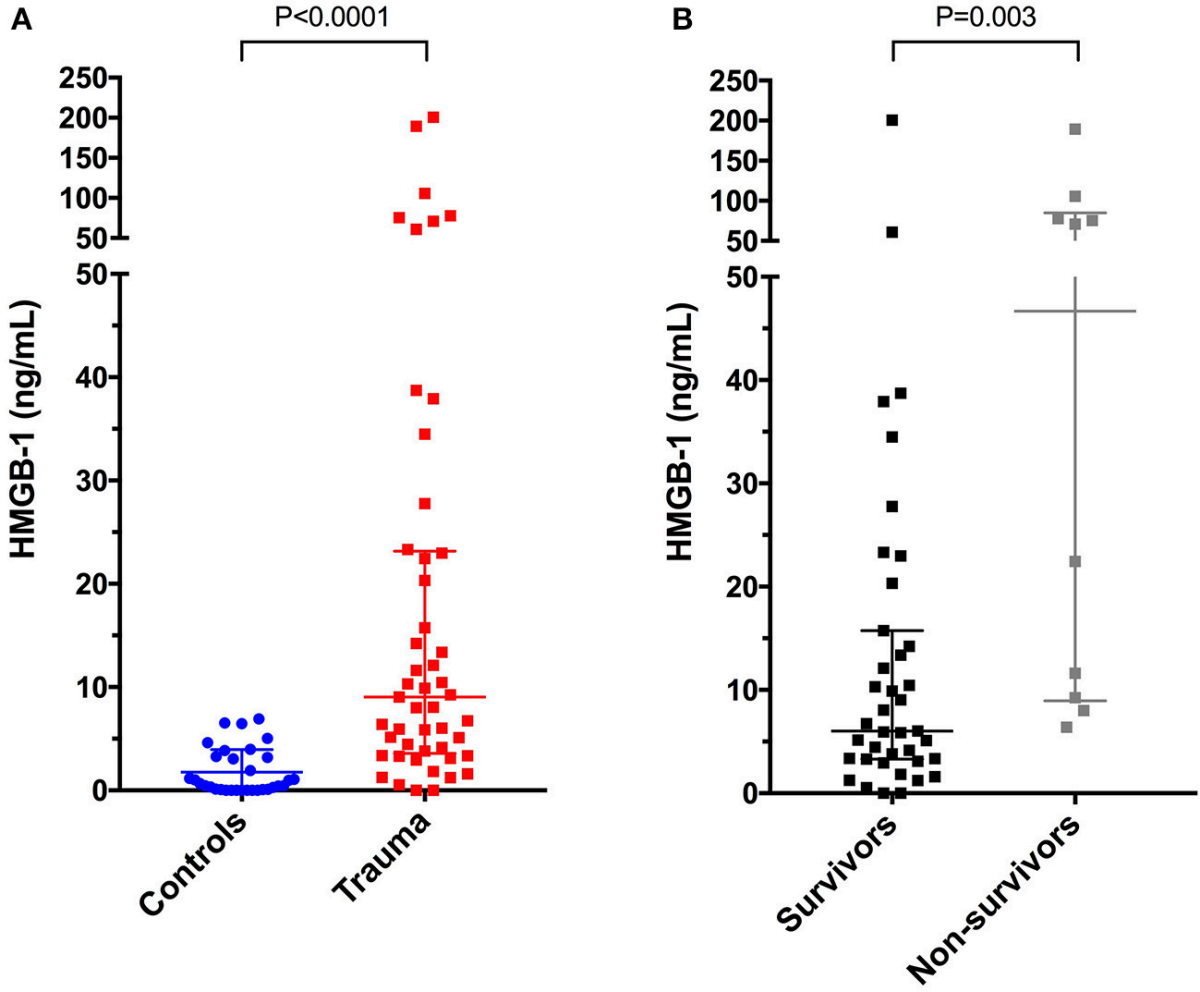
Scatterplots of plasma high-mobility group box-1 (HMGB-1) in **(A)** healthy controls (*n* = 33) compared to dogs following moderate-severe trauma (*n* = 49), and in **(B)** survivors (*n* = 39) compared to non-survivors (*n* = 10). The central horizontal line represents the median, and the two error bars represent the interquartile range. Comparisons between the groups were performed using the Mann-Whitney U test. *P* < 0.05 was considered significant.

**Figure 2:**
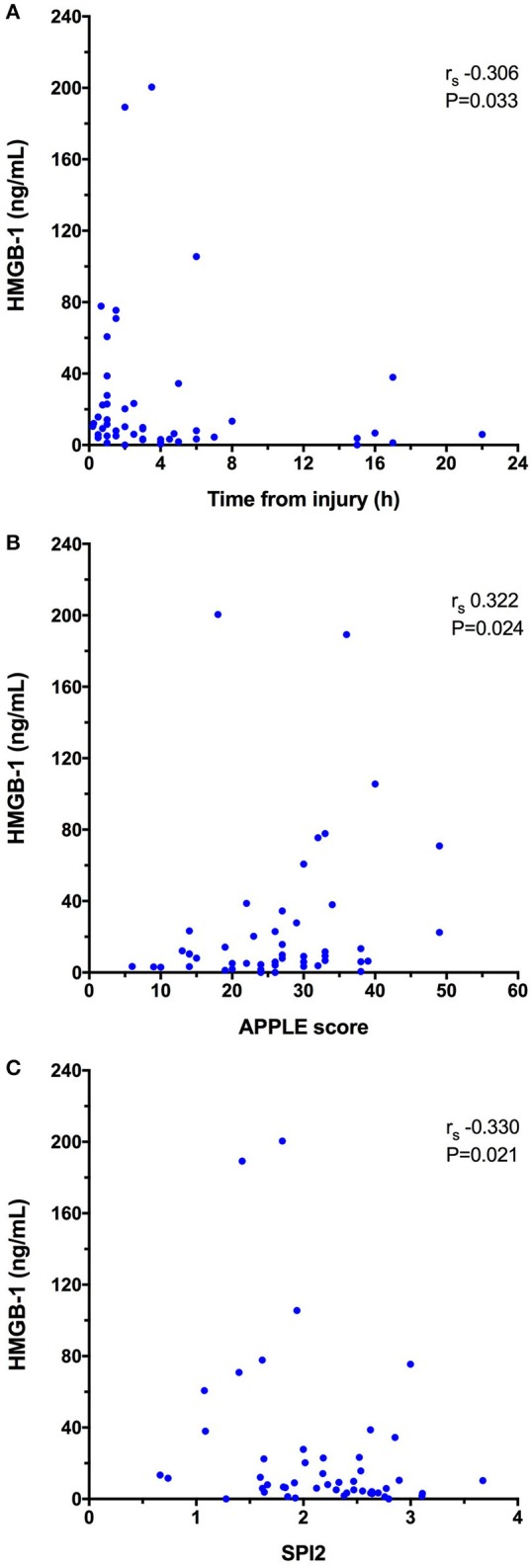
X-Y scatterplots of high-mobility group box-1 (HMGB-1) concentrations against **(A)** time from injury to presentation (h), **(B)** Acute Patient Physiology and Laboratory Evaluation (APPLE) score, **(C)** Survival Prediction Index-2 (SPI2). Spearman's correlation coefficients (r_s_) with associated *P*-values are displayed in each panel. *P* < 0.05 was considered significant.

HMGB-1 concentrations (Figure 1A) were significantly greater in dogs following trauma 9.03 ng/mL (3.59–23.15) compared to healthy controls 0.56 ng/mL (0.08–3.25), (*P* < 0.0001). In dogs following trauma, HMGB-1 concentrations were significantly greater in non-survivors 46.67 ng/mL (8.94–84.73) compared to survivors 6.03 ng/mL (3.30–15.75), (*P* = 0.003) (Figure 1B). Excluding the four dogs with hemolysis altered the corresponding *P*-values but did not impact the result of either of these two analyses. HMGB-1 concentrations were not associated with the mechanism of trauma (i.e., blunt trauma vs. other causes e.g., bite injuries) but were significantly greater in dogs that presented as primary walk-in emergencies 11.86 ng/mL (5.69–44.22) compared to those that were referred from a primary care veterinarian 5.94 ng/mL (2.94–13.37), (*P* = 0.024). Multivariable logistic regression analysis suggested that the association between HMGB-1 concentrations and survival to discharge remained after accounting for prior assessment by a primary care veterinarian (Supplementary Table 2). There was a mild but significant negative correlation between time from trauma and HMGB-1 concentration (r_s_ −0.306, *P* = 0.033). HMGB-1 was positively correlated with APPLE score (r_s_ 0.322, *P* = 0.024) and negatively correlated with SPI2 (r_s_ −0.330, *P* = 0.021) (Figures 2A–C), but there was no significant correlation between ATT score and HMGB-1 concentrations.

### Cytokines (bead-based assays)

**Figure 3:**
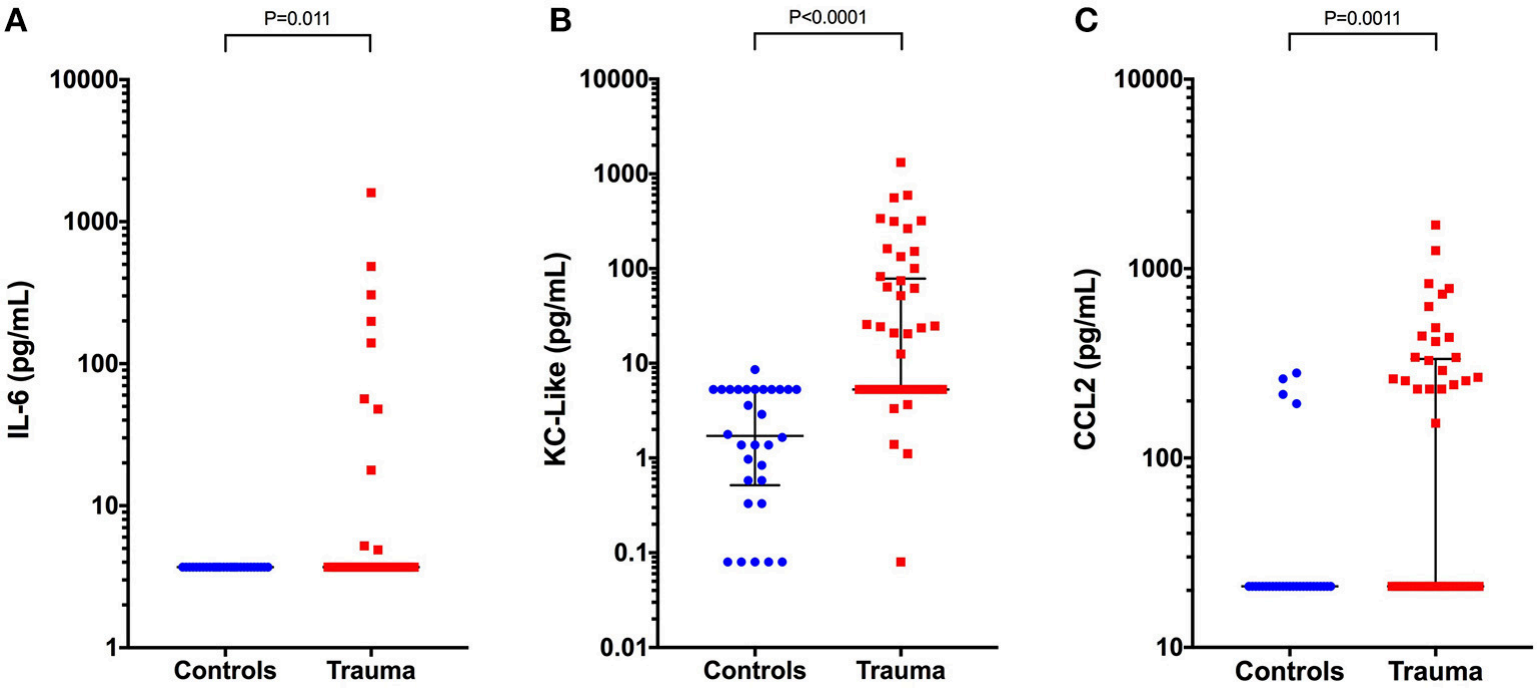
Scatterplots of plasma cytokine concentrations measured using a bead-based multiplex assay in healthy controls (*n* = 33) compared to dogs following moderate-severe trauma (*n* = 49). **(A)** Interleukin-6 (IL-6) concentrations, **(B)** keratinocyte-chemoattractant-like (KC-Like), and **(C)** C-C motif chemokine ligand 2 (CCL2). Comparisons between the groups were performed using the Mann-Whitney U test. *P* < 0.05 was considered significant.

**Figure 4:**
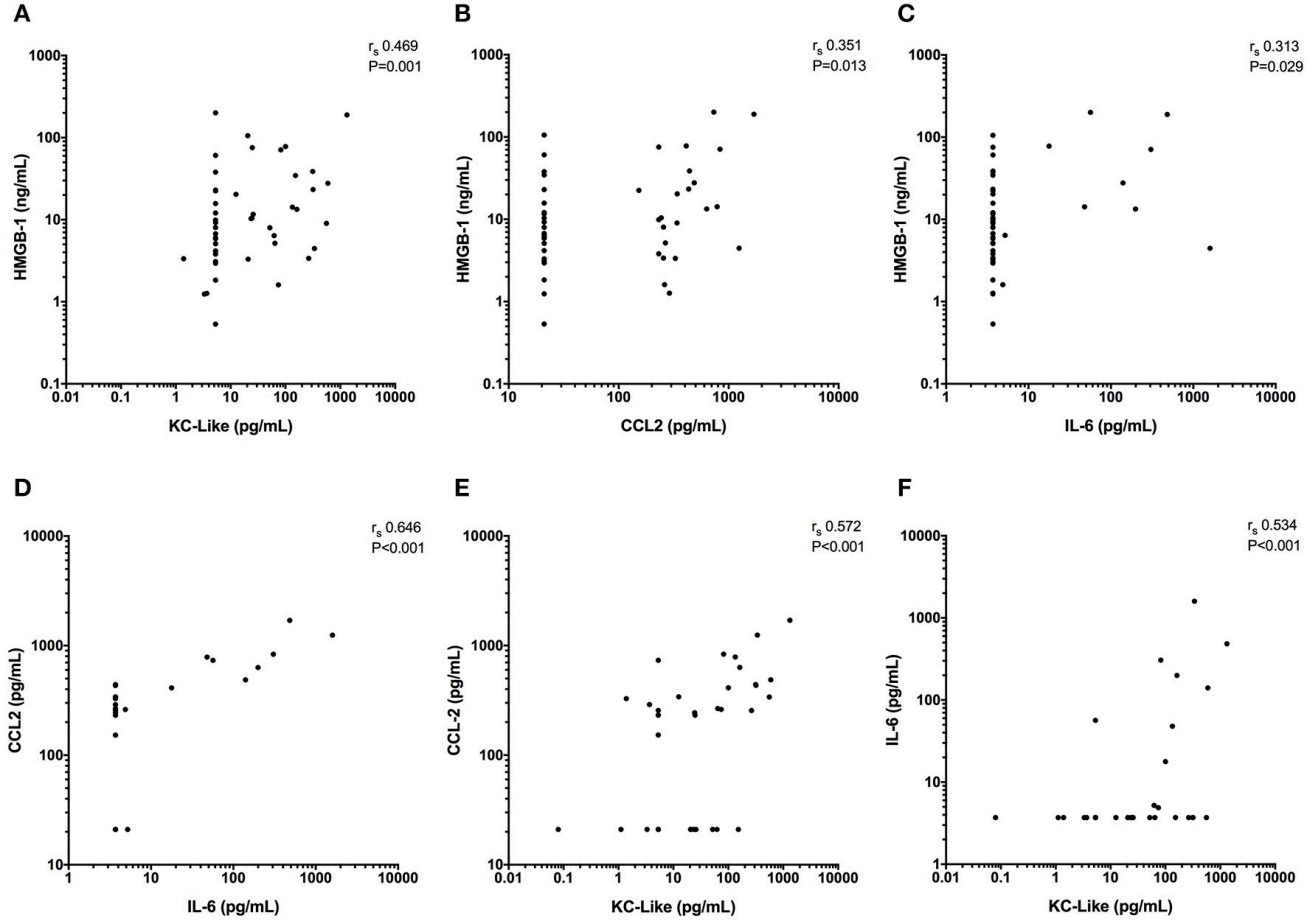
X-Y scatterplots of high mobility group box-1 (HMGB-1) and various cytokines against each other. **(A–C)** HMGB-1 concentrations compared to concentrations of keratinocyte chemoattractant-like (KC-Like), C-C motif chemokine ligand 2 (CCL2) and interleukin-6 (IL-6). **(D, E)** CCL2 vs. IL-6 and KC-Like and **(F)** IL-6 against KC-Like. Spearman's correlation coefficients (r_s_) with associated *P*-values are displayed in each panel. *P* < 0.05 was considered significant.

Concentrations of IL-6, KC-like, and CCL2 were significantly greater in injured dogs compared to healthy controls (all *P* ≤ 0.011) (Figure 3). None of these cytokines as measured using the multiplex bead-based assay were predictive of survival to discharge (Table 3). Likewise, none of the cytokines measured by the bead-based assay were significantly correlated with any of the injury or illness severity scores and were not different between different mechanisms of trauma. There were no significant differences in cytokine concentrations measured with the bead-based assay between walk-in cases and those referred (all *P* ≥ 0.163). HMGB-1 was positively correlated with KC-Like (r_s_ 0.469, *P* = 0.001), CCL2 (r_s_ 0.351, *P* = 0.013), and IL-6 (r_s_ 0.313, *P* = 0.029) (Figures 4A–C). There were moderate or moderately strong positive correlations between IL-6 and CCL2 (r_s_ 0.646, *P* < 0.001), KC-Like and CCL2 (r_s_ 0.572, *P* < 0.001) and between KC-Like and IL-6 (r_s_ 0.534, *P* < 0.001) (Figures 4D–F).

**Table 3:** Cytokine concentrations in dogs following moderate-severe trauma that survived to hospital discharge compared to dogs with trauma that did not survive to hospital discharge; data are presented as median (interquartile range).

**Analysis method**	**Cytokine**	**Survivors (*n* = 39)**	**Non-survivors (*n* = 10)**	***P*-value**
Canine-specific ELISA	IL-2 (pg/mL)	4.7 (2.7–7.6)	7.07 (1.2–24.7)	0.645
	IL-6 (pg/mL)	50.1 (36.8–124.0)	132.7 (54.1–389.1)	0.054
	CXCL8 (pg/mL)	474.7 (207.9–996.0)	698.3 (318.9–2147.0)	0.094
	TNF-α (pg/mL)	0.17 (0.06–0.37)	0.45 (0.05–0.77)	0.184
Bead-based Multiplex Assay	IL-6 (pg/mL)	3.7 (3.7–3.7)	3.7 (3.7–89.8)	0.1425
	CXCL8 (pg/mL)	127.8 (21.7–483.1)	245.7 (167.5–885.4)	0.108
	KC-Like (pg/mL)	5.3 (5.3–74.4)	38.8 (16.8–86.8)	0.072
	CCL2 (pg/mL)	21 (21–327.7)	86.8 (21–517.3)	0.807

*P-values were calculated using the Mann-Whitney U test. P < 0.05 was considered significant. CCL2, C-C motif chemokine ligand 2; CXCL8, C-X-C motif chemokine 8; IL-2, interleukin-2; IL-6, interleukin-6; KC-Like, keratinocyte chemoattractant-like; TNF-α, tumor necrosis factor alpha*.

### Cytokines (ELISA)

**Figure 5:**
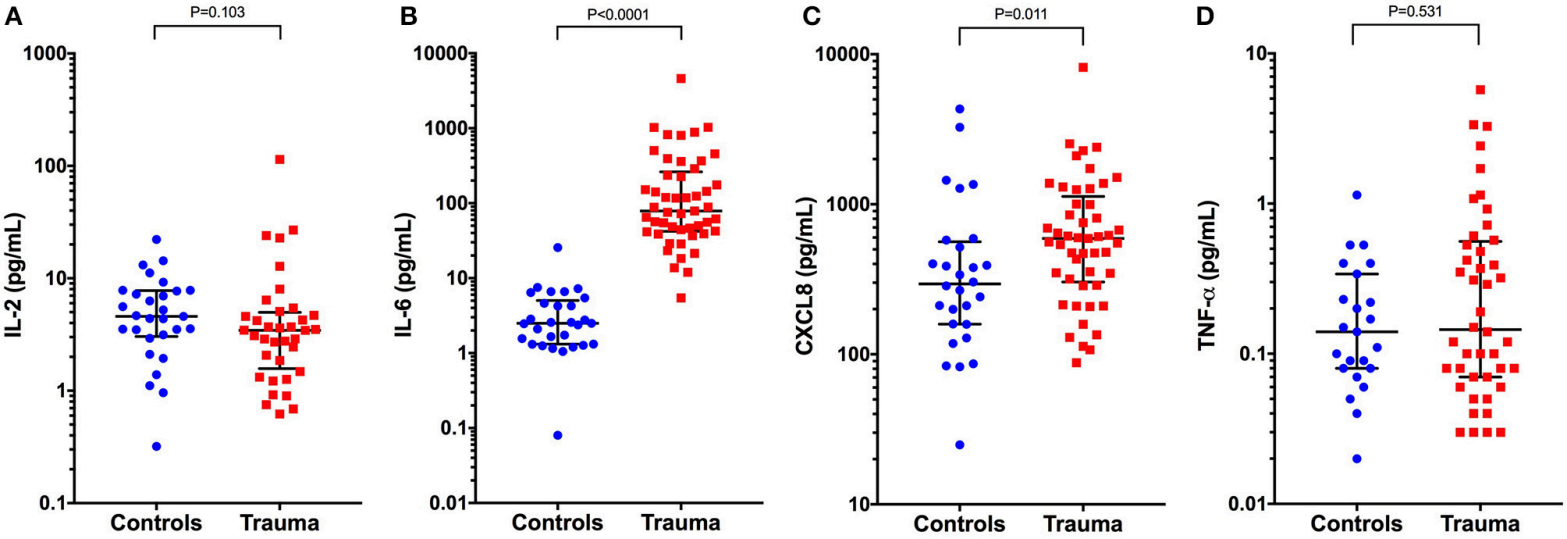
Scatterplots of plasma cytokine concentrations measured using a canine-specific ELISA in healthy controls (*n* = 33) compared to dogs following moderate-severe trauma (*n* = 49). **(A)** Interleukin-2 (IL-2) concentrations, **(B)** IL-6, **(C)** C-X-C motif chemokine 8 (CXCL8), and **(D)** Tumor necrosis factor alpha (TNF-α). Comparisons between the groups were performed using the Mann-Whitney U test. *P* < 0.05 was considered significant. The central horizontal line represents the median, and the two error bars represent the interquartile range. Comparisons between the groups were performed using the Mann-Whitney U test. *P* < 0.05 was considered significant.

Concentrations of IL-6 and CXCL8 were significantly greater in injured dogs compared to healthy controls (all *P* ≤ 0.011) (Figure 5). As with the bead-based assay, none of the cytokines measured by canine specific ELISA were predictive of survival to discharge (Table 3). None of the cytokines measured by the canine-specific ELISA were significantly correlated with any injury or illness severity scores or with HMGB-1 and were not different between different mechanisms of trauma. There were no significant differences in cytokine concentrations measured with the ELISA assay between walk-in cases and those referred (all *P* ≥ 0.267). There was a moderately-strong positive correlation between IL-2 and CXCL8 (r_s_ 0.626, *P* < 0.001) (Figure S1). Of the cytokines measured by two different methods (IL-2, IL-6, CXCL8, and TNF-α), only the values for CXCL8 were positively correlated with each other (r_s_ 0.331, *P* = 0.020) (Figure S2).

### Survival prediction

From the available biomarkers including HMGB-1 and the cytokines, 4 variables were associated with survival to discharge at *P* < 0.1 (HMGB-1, IL-6, CXCL8, and KC-like). There was no association between the shock index and survival. None of these variables exhibited collinearity and hence all were considered as candidate outcome predictors. The ATT, APPLE, MGCS, SOFA, and SPI2 scores were also all associated with survival to discharge at *P* < 0.1. Of these, only the most discriminant illness severity scoring system (APPLE) was inputted as a possible candidate predictor of survival to discharge. The 4 biomarkers and the APPLE score were entered into a survival multivariable analysis in a forward stepwise fashion and retained in the final model if significantly (*P* < 0.05) associated with survival to discharge. The final model contained only the APPLE score, as an independent predictor of non-survival (OR 1.30, 95% CI 1.09–1.55, *P* = 0.003). The equation of the model was log(OR) = 0.264 × APPLE score −9.346. The Hosmer-Lemeshow chi-square value for this model was 4.26 with a non-significant *P* = 0.833, indicating that the model was adequately fitted. The Nagelkerke *R*^2^ value was 0.535, indicating that the model explained approximately 54% of the variability in the data. Construction of receiver operating characteristic (ROC) curves and calculation of the Youden index identified that a cut-off for the APPLE score of 31, was 90% sensitive and 84.6% specific for non-survival (AUROC = 0.912, *P* < 0.001) (Figure S3).

## Discussion

The present study identified significantly increased concentrations of HMGB-1 in dogs following trauma and determined that HMGB-1 was associated with non-survival. There was some overlap between healthy control values and those in dogs following trauma. The positive correlation of HMGB-1 with APPLE score suggests that HMGB-1 may be better used to gauge injury severity following trauma, although surprisingly HMGB-1 did not correlate with ATT score. This may be due to the timing of sample collection or perhaps reflects the nature of the injuries in dogs compared with common patterns of injury in people.

Multiple studies have now evaluated blood HMGB-1 concentrations in dogs with GDV, pyometra and sepsis (17, 40–42). Concentrations of HMGB-1 are reportedly prognostic in dogs with GDV (17) and in dogs with systemic inflammation (40, 41). Other studies have not confirmed the prognostic value of HMGB-1 in GDV syndrome (43) or sepsis however (19), suggesting that the value of this biomarker varies with the population, the illness severity and the nature of the disease process. In the present study, all dogs were assessed following trauma, but the nature, severity and timing of these injuries varied. These sources of variation might have affected the association between HMGB-1 and outcome in the present study. Evaluation of HMGB-1 concentrations at fixed time intervals after a standardized, experimental injury would enable the relationship between HMGB-1 concentrations and survival to be most accurately identified.

In severely injured people, HMGB-1 is an early pro-inflammatory mediator that peaks between 2 and 6 h post-trauma (44, 45). In contrast, it should be noted that HMGB-1 concentrations peak at 72 h in dogs following ovariohysterectomy (41), and also increase post-operatively in dogs with pyometra (42). In dogs with GDV, HMGB-1 concentrations increased up to 22 h post-surgery (17). Hence the value of HMGB-1 may be dependent on the timing of measurement. In future studies, serial analyses will be necessary to identify the optimal timing of HMGB-1 measurement in canine trauma. The dynamics of HMGB-1 concentrations in diverse patient populations including sepsis, SIRS, pyometra, GDV, trauma, and routine surgery likely reflect a complex interplay of disease process, illness severity, clinician interventions, and measurement timing. The veterinary HMGB-1 literature encompasses various studies of distinct disease entities. Measured concentrations and the timing of peak HMGB-1 levels likely varies with the degree of tissue injury, pathogen-induced release and clinician intervention. As such, it may be challenging to directly compare HMGB-1 concentrations and kinetics across disease processes.

Irrespective of its value for prognostication, measurements of HMGB-1 may be of value in the future for guiding therapy. Neutralization of HMGB1 is protective against tissue damage in various preclinical models of inflammatory diseases (11), although human trials targeting HMGB-1 have not been performed to date. A recent experimental swine study suggests that fluid resuscitation practice may influence the release of DAMPs following trauma. In pigs with combined traumatic brain injury and hemorrhagic shock, resuscitation with fresh frozen plasma attenuated release of nucleosomes and prevented depletion of DNAse I compared to normal saline (46). Similarly, in rodent traumatic brain injury models, anti-HMGB1 monoclonal antibodies may protect against disruption of the blood-brain barrier and reduce HMGB-1 induced inflammation (47). It remains to be determined whether interventions targeted at HMGB-1 will be beneficial in canine trauma, but further investigation appears warranted.

The cytokine profile of dogs with trauma in the present study was pro-inflammatory (22, 25, 27, 48), with concentrations of IL-6, CXCL8, KC-like, and CCL2 all increased compared to healthy controls. The findings of the present study are similar to those in human trauma (10, 32, 49–51). Simultaneous measurements of multiple cytokines in people have found non-parametric distributions of cytokine concentrations, as was the case here. Human studies report many patients have concentrations at the lower limits of detection, with log-scale differences between the lowest and highest concentrations. Human studies also report strong correlations between chemokine concentrations (52). All of these findings match those from the present study. We speculate that these log-scale differences exist because of transient positive feedback associated with transcription factor activation by TLR signaling (53, 54). It seems probable that the correlations between cytokines exist because of a common stimulus for their release and the shared signaling pathways downstream of the Toll-like receptors (55, 56). In people, concentrations of IL-6, CXCL8, and CCL2 are frequently correlated with the degree of injury (10, 57, 58). This was not the case in the present study, where only HMGB-1 was correlated with injury severity in dogs as indicated by APPLE score.

The findings of the present study are comparable to a recent study of dogs with systemic inflammatory response syndrome that included 6 dogs following trauma (30). That study reported that IL-6 concentrations measured at presentation and prior to therapy were significantly increased compared to samples collected from the same dogs 1 month after hospital discharge, but no significant change in IL-6 concentrations was detected during the first 72 h of hospitalization. Using a different assay to those employed in the present study, Gommeren and others detected TNF-α in only 29% dogs at presentation, while none of the dogs in the study had detectable TNF-α concentrations 1-month post-discharge (30). Neither IL-6 or TNF-α were discriminating for the cause of systemic inflammation or predictive of survival in that study.

The present study identified several positive correlations between various cytokines including IL-6, KC-Like, and CCL2. To an extent this is to be expected since similar cell types produce these cytokines in response to identification of pathogen- or damage-associated molecular patterns (22). We speculate that similar tissue injury stimuli led to the production of these cytokines. The clinical relevance of these correlations may be limited, however.

In a study on cytokines in dogs with sepsis and IMHA, the authors grouped cytokines into 4 categories in order to enable comparisons between disease processes (22). Cytokines were grouped into chemokines (CCL2, KC-like, CXCL8, IP-10), pro-inflammatory cytokines (IL-6, IL-18), anti-inflammatory cytokines (TGFβ, IL-10), and T-cell derived cytokines (IL-2, IL-4, IL-7, IL-15). A similar approach was not adopted here because not all of these cytokines were measurable in the present study population and it was not felt appropriate to generate alternate arbitrary groups. We sought to determine if the concentrations of individual cytokines were associated with survival in the present study. While more detailed insights might be available from panels of cytokines, a single analyte that enables prognostication would be more valuable, cheaper and more expedient from a clinical perspective.

An anti-inflammatory pattern of cytokine activity was not identified in the present study, which may be due to patient or to assay factors. Trauma in dogs may be predominantly pro-inflammatory, or more likely, the dogs in the present study may have been sampled at times when pro-inflammatory cytokines predominated, consistent with the first-hit concept of major trauma (59). This is plausible because all dogs were sampled at presentation and although there was some variation in the time interval from trauma to presentation, most dogs were sampled within 2.5 h of trauma. As such, it may be that sampling these dogs at other time points might have identified an anti-inflammatory profile or a mixed pattern.

Approximately half of the dogs were assessed by a primary care veterinarian prior to sampling for this study, but there was no difference in any of the cytokine concentrations between dogs seen as a walk-in emergency compared to those seen on a referral basis. Our data therefore suggest that the initial response to trauma is pro-inflammatory, but it is noteworthy that the degree of inflammation did not correspond with survival. This suggests that this pro-inflammatory phase following trauma is likely appropriate, physiologic, and adaptive. In this respect our data are consistent with the findings of Gommeren et al. (30) who found no association between the concentrations of IL-6 or TNF-α and survival in their population of dogs with SIRS that included 6 dogs following trauma. The data from the present study are distinct from a recent study we conducted in canine sepsis; however, where the concentration of CCL2 was highly discriminant for non-survival (19). This may be due to fundamental differences in the pathophysiology of sepsis due to bacterial infection where chemokine concentrations may reflect the degree of pathogen invasion and trauma, where tissue damage is the predominant signal initiating cytokine secretion.

The lack of a significant difference between the TNF-α concentrations in healthy controls and dogs following trauma is, at first inspection, surprising. Although TNF-α is well-recognized to be an early pro-inflammatory cytokine, TNF-α is predominantly cell-associated, rather than secreted (60). Increased plasma concentrations do occur in human trauma (32, 57), but in only 40% of cases in some populations (61), and the timing of sampling may also affect identification of increased TNF-α concentrations. Plasma TNF-α concentrations may reflect cell-expression, the activity of metalloproteinase enzymes and the concentration of shed TNF receptors (62, 63). It is also possible the multiplex assay that was employed was relatively insensitive to small changes in cytokine concentrations. Although this assay has been widely used, some analyte concentrations are frequently below the limits of detection (31, 64–66), as was the case in the present study.

We recognize our study has limitations. Biomarker concentrations were measured only at enrollment. The kinetics of the biomarkers measured are dynamic and are likely distinct between the different markers. As such, it is likely the performance of these biomarkers for the assessment of dogs following trauma was not optimized. Serial evaluation of biomarkers in response to therapy may better define the roles of these biomarkers in prediction of complications such as infection or disseminated intravascular coagulation, the need for repeated surgery or the development of secondary organ dysfunction. The data presented here are likely reflective of the heterogeneity that exists in trauma syndromes in other populations, but differences in populations, and the type and extent of injuries sustained by dogs in the present study, may not match other populations and hence limit the generalizability of our results. The ATT score was chosen as an inclusion criterion in order to identify a population of dogs with moderate-severe trauma. This score was chosen because of its ease of use, lack of requirement for any laboratory testing and because it has been documented to be associated with survival in several publications (3, 33). The ATT score therefore is likely to be an accurate gauge of injury severity and hence it was considered reasonable to evaluate the correlation between biomarker concentrations and the ATT score as a proxy for injury severity. Use of the ATT score as an entry criterion might have positively biased these correlations. However, this did not appear to be the case because few of the biomarker concentrations were correlated with ATT score.

This study involved two distinct patient populations, namely those that had been seen by a primary care veterinarian before referral and those cases that were presented to our institution as walk-in emergencies. Many of the patients that were referred to us received some therapy prior to sample collection for this study. This prior therapy may have altered the measured concentrations of the biomarkers in unpredictable ways and hence may have affected the relationships between parameters that we assessed. Using an approach similar to that of Gommeren et al. (30), who sampled all patients prior to having received any therapy would eliminate this potential source of bias, but at our institution would have significantly increased the time taken to recruit all of the cases for this study.

The present study used stored samples that had been frozen for up to 15 months prior to analysis. For practical and financial reasons, the interval between sampling and analysis could not be standardized and hence this could have impacted the results in unpredictable ways. The stability of canine cytokine concentrations in samples frozen at −80°C is not known, however, human IL-6 is stable for several years when frozen at −70°C (67), which suggests that the time of storage had limited, if any, impact on our findings. The limited correlation between cytokine concentrations measured with the two different assays is of concern and is hard to explain. No gold standard for cytokine measurement exists in veterinary medicine, but both assays employed here have been previously reported by various groups (19, 22, 30, 31, 38, 68–70). Further investigation and comparison of these two methods appears warranted. However, an analysis to determine the precision and accuracy of these two assays, with samples of known cytokine concentration, was outside of the current study. Irrespective, the limited agreement identified argues that the same cytokine assay should be used consistently whenever serial analyses are performed.

In the present study, multiple analyses were performed on various biomarkers in a comparatively small population of dogs. Although the analyses performed were based on data-driven *a priori* hypotheses regarding biomarker concentrations in dogs following trauma, there remains an increased likelihood of a false positive identification of differences that are not real. The best method to address this situation would be to increase the sample size. This can be challenging in veterinary medicine due to cost and caseload limitations, although a multicenter approach might help to overcome some of these issues. An alternative method for *post-hoc* correction for multiple comparisons, the Bonferroni correction, is very conservative and would likely have incorrectly eliminated some of the reported findings. Given that it was hypothesized *a priori* that there would be differences in cytokine concentrations between groups, the Bonferroni correction was not deemed appropriate and was not applied.

In summary, the present study demonstrates that dogs following moderate-severe trauma have significantly increased concentrations of HMGB-1 and pro-inflammatory cytokines compared to healthy controls. None of the cytokines measured were associated with survival to discharge in this cohort. Plasma concentrations of the damage-associated molecular pattern molecule HMGB-1 were prognostic but were not predictive of survival to discharge independent of illness severity as adjudicated by the APPLE score. This suggests that HMGB-1 may not provide information in addition to that captured by a comprehensive illness severity score. It could be argued that the cost and time required for its measurement may not be justified in routine clinical practice, but we did not optimize the sampling time for, or perform serial analysis of, HMGB-1. The ideal biomarker tracks the course of recovery following trauma and may predict other outcome measures such as organ dysfunction, development of infection or other complications not studied in the present study. Hence, sample time optimization, serial sampling studies, and evaluations of other outcome measures are warranted to determine how to maximize the value of HMGB-1 measurement in the assessment and management of the trauma patient.

## Ethics statement

All samples analyzed in this study were collected from dogs managed at the institution veterinary teaching hospital (Cornell University, Ithaca, NY) as part of studies approved by the local Institutional Animal Care and Use Committee (IACUC), and undertaken under written informed client consent (Cornell IACUC 2014-0053). Healthy privately-owned dogs were enrolled as controls with local Institutional Animal Care and Use Committees approval and written informed client consent (Cornell IACUC 2014-0052).

## Author contributions

RG designed the study, collected and analyzed data and co-wrote the manuscript. J-AL assisted with study design, collected and analyzed data and co-wrote the manuscript. Both authors contributed to, read and approved the final manuscript.

### Conflict of interest statement

The authors declare that the research was conducted in the absence of any commercial or financial relationships that could be construed as a potential conflict of interest.

## Supplementary material

Figure S1 X-Y scatterplot of CXCL8 concentrations measured with a canine specific ELISA assay against concentrations of IL-2 concentrations from 49 dogs after moderate-severe trauma. Spearman's correlation coefficient (r_s_) with the associated *P*-value is displayed; *P* < 0.05 was considered significant.

Figure S2 X-Y scatterplot of CXCL8 concentrations measured with a canine specific ELISA assay (abscissa) against CXCL8 concentrations measured using a bead-based multiplex assay (ordinate) from 49 dogs after moderate-severe trauma. Spearman's correlation coefficient (r_s_) with the associated *P*-value is displayed; *P* < 0.05 was considered significant.

Figure S3 A receiver operating characteristic (ROC) curve of Acute Patient Physiology and Laboratory Evaluation (APPLE) score for the prediction of survival in 49 dogs following moderate-severe trauma. The area under the curve (AUC) was 0.912, which was significantly >0.5, (*P* < 0.0001).

Data Sheet 1 Summaries of the illness severity scores used in the present study, including their derivation and calculation.

Supplementary Table 1 Summary statistics for complete blood count and serum chemistry panels collected at presentation to the study institution. Data are presented in SI units. Institution reference intervals are indicated in parentheses.

Supplementary Table 2 Summary of multivariable logistic regression model assessing the association between HMGB-1 concentrations and survival to discharge accounting for prior assessment by a primary care veterinarian.

## Abbreviations

APPLE: acute patient physiologic and laboratory evaluation
AUROC: area under the ROC curve
CCL: C-C motif chemokine ligand
CXCL: C-X-C motif chemokine
DAMPs: damage associated molecular patterns
GM-CSF: granulocyte macrophage-colony stimulating factor
HMGB-1: high-mobility group box-1
IL: interleukin
IFN: interferon
IMHA: immune-mediated hemolytic anemia
KC-Like: keratinocyte-derived chemokine-like
LPS: lipopolysaccharide
MGCS: modified Glasgow coma scale score
OR: odds ratio
ROC: receiver operating characteristic
SIRS: systemic inflammatory response syndrome
SOFA: sequential organ failure assessment
SPI2: survival prediction index-2
SpO2: oxygenation saturation
TNF: tumor necrosis factor.
