# Supplementary figures and images for: High Mobility Group Box-1 and Pro-inflammatory Cytokines Are Increased in Dogs After Trauma but Do Not Predict Survival
Source: Front Vet Sci. 2018 Jul 30;5:179. doi: 10.3389/fvets.2018.00179 (PMC6077187; doi:10.3389/fvets.2018.00179)

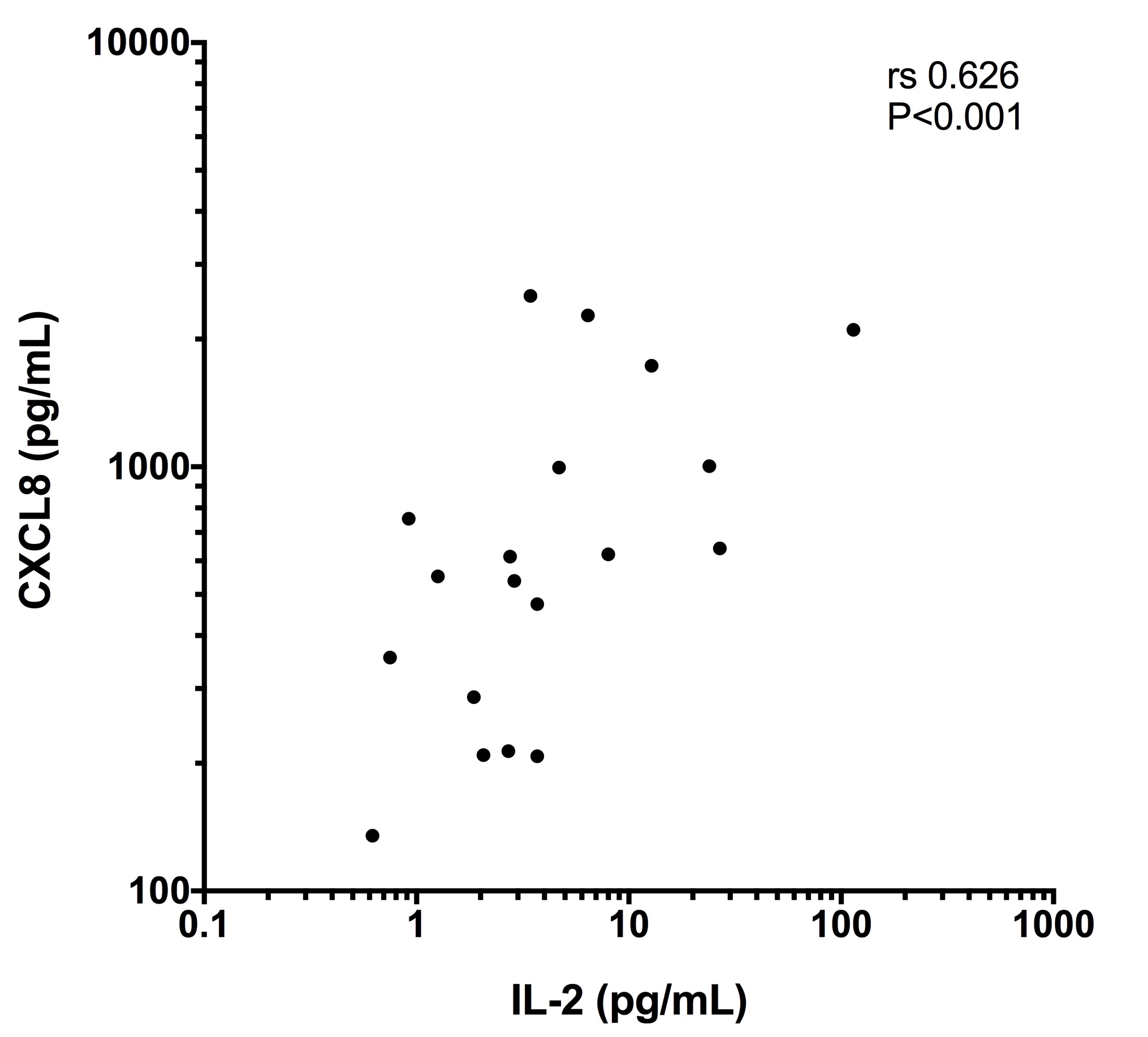

Supplement: Figure S1 — X-Y scatterplot of CXCL8 concentrations measured with a canine specific ELISA assay against concentrations of IL-2 concentrations from 49 dogs after moderate-severe trauma. Spearman's correlation coefficient (rs) with the associated P-value is displayed; P < 0.05 was considered significant. [file Image_1.JPEG]

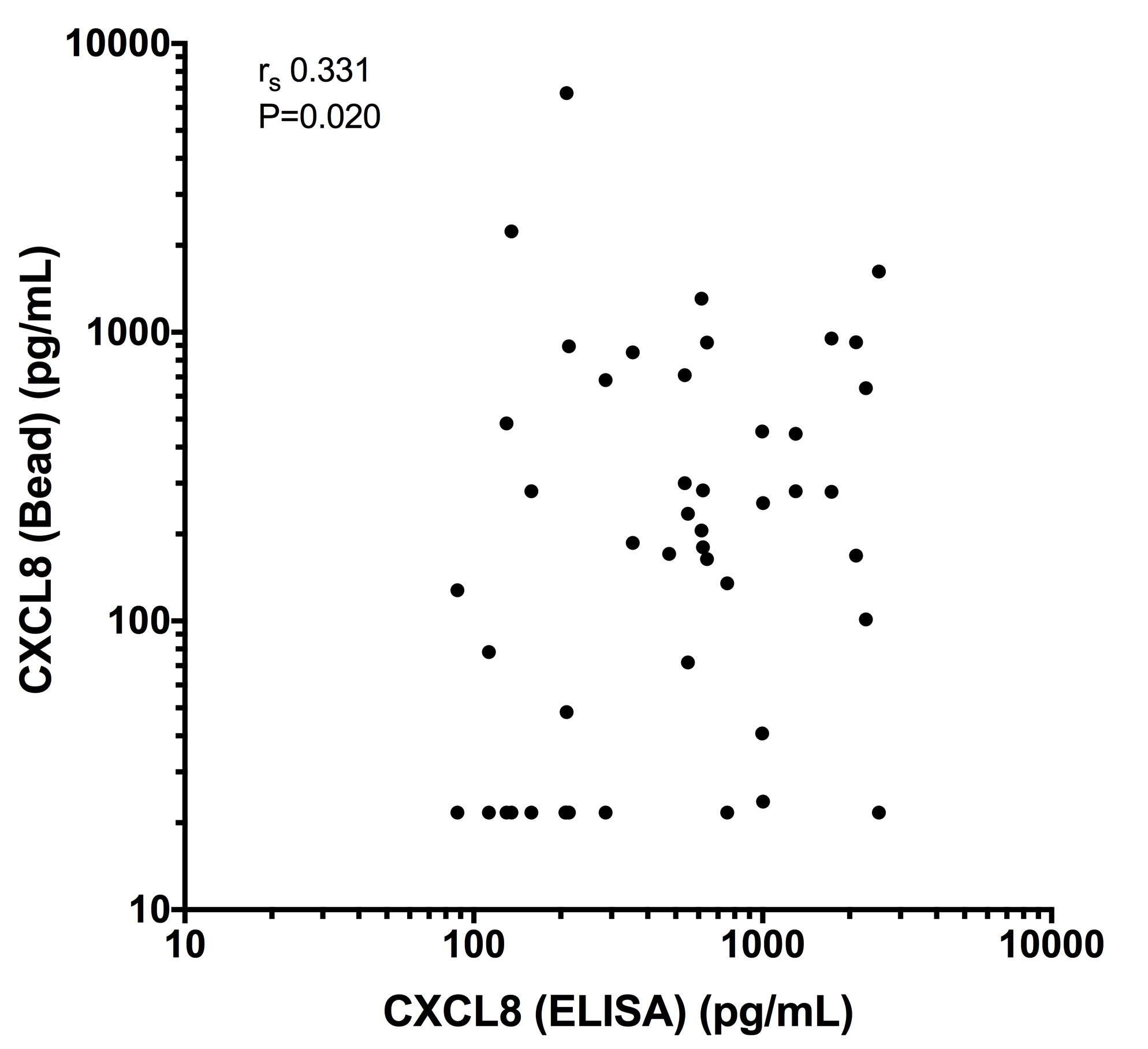

Supplement: Figure S2 — X-Y scatterplot of CXCL8 concentrations measured with a canine specific ELISA assay (abscissa) against CXCL8 concentrations measured using a bead-based multiplex assay (ordinate) from 49 dogs after moderate-severe trauma. Spearman's correlation coefficient (rs) with the associated P-value is displayed; P < 0.05 was considered significant. [file Image_2.JPEG]

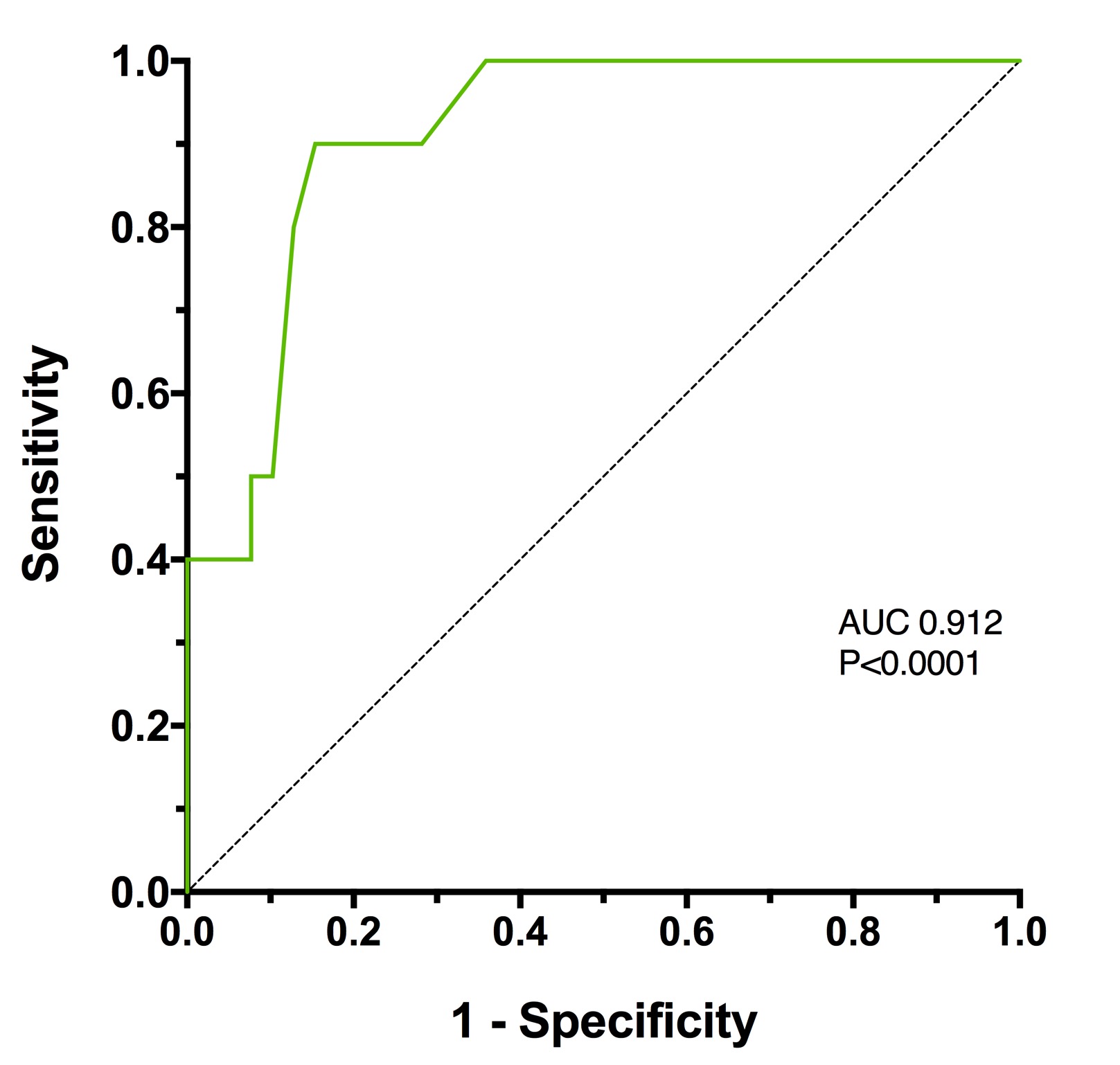

Supplement: Figure S3 — A receiver operating characteristic (ROC) curve of Acute Patient Physiology and Laboratory Evaluation (APPLE) score for the prediction of survival in 49 dogs following moderate-severe trauma. The area under the curve (AUC) was 0.912, which was significantly >0.5, (P < 0.0001). [file Image_3.JPEG]
