# Supplementary material for: High Mobility Group Box-1 and Pro-inflammatory Cytokines Are Increased in Dogs After Trauma but Do Not Predict Survival
Source: Front Vet Sci. 2018 Jul 30;5:179. doi: 10.3389/fvets.2018.00179 (PMC6077187; doi:10.3389/fvets.2018.00179)
Supplement: Data Sheet 1 — Summaries of the illness severity scores used in the present study, including their derivation and calculation. [file Data_Sheet_1.DOCX]

**Summaries of illness and injury scores**

**Acute Patient Physiologic and Laboratory Evaluation score (APPLE)**

The canine APPLE score (Hayes et al. 2010) involves assigning point values to various physiologic and laboratory abnormalities according to the following table. The APPLE score is the sum of these values.


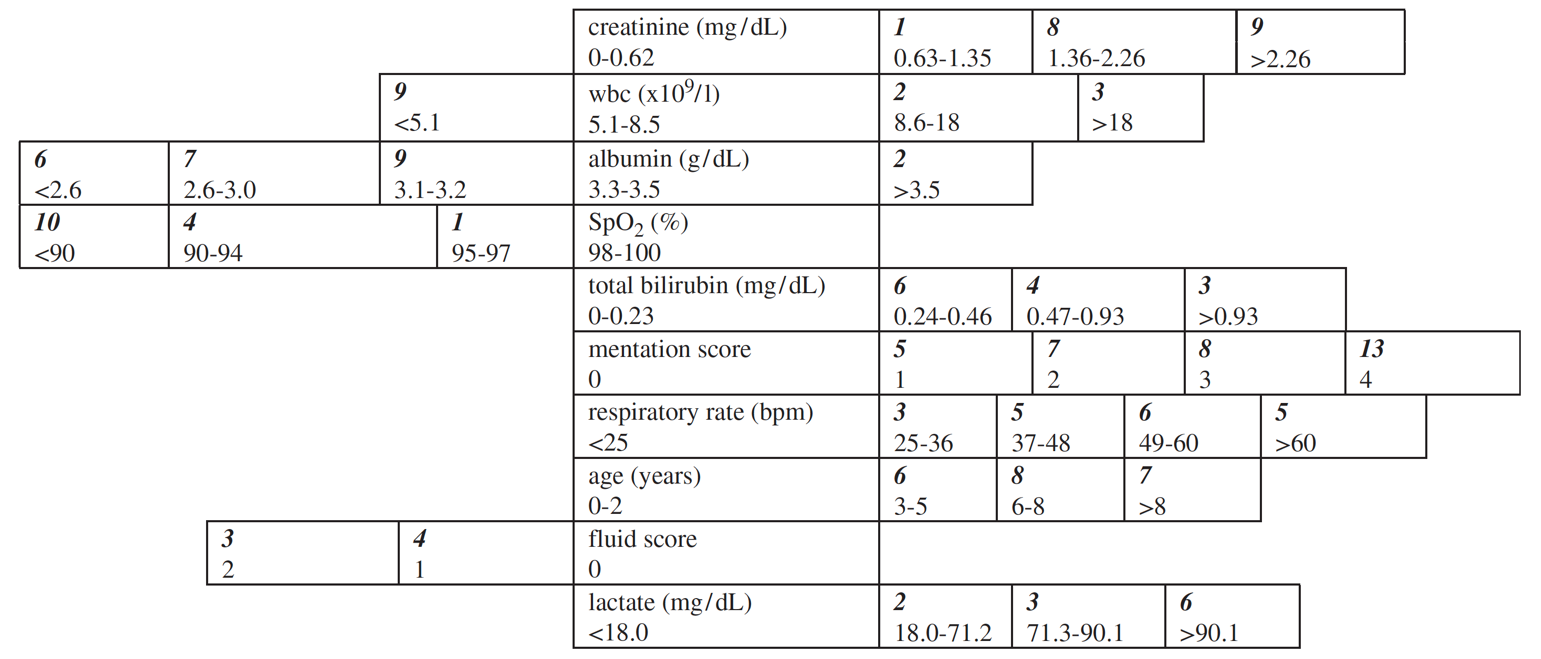


**Rapid Acute Patient Physiologic and Laboratory Evaluation score (APPLE_fast_)**

The shortened version of the full canine APPLE score (Hayes et al. 2010) is similar to the full score but requires measurement of fewer variables. The APPLE_fast_ score is the sum of the values assigned per the table below.


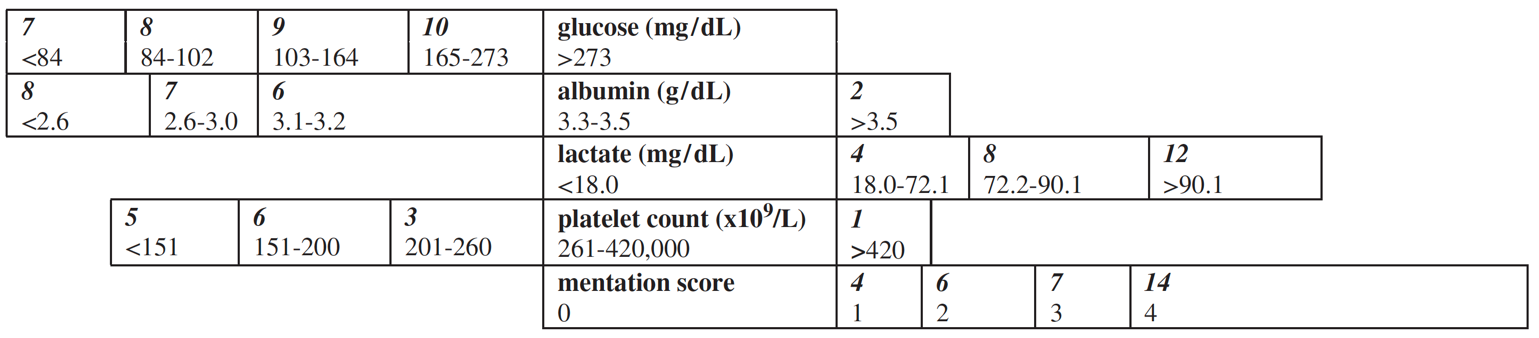


**Animal Trauma Triage score (ATT)**

The ATT score was developed to enable rapid initial assessment of canine and feline trauma patients (Rockar et al. 1994). It involves assigning a score of 0-3 in each of 6 categories per the table below. The ATT is the sum of these individual scores.

| **Grade** | **Perfusion** | **Cardiac** | **Respiratory** | **Eye/Muscle/Skin** | **Skeletal** | **Neurological** |
| --- | --- | --- | --- | --- | --- | --- |
| 0 | • MM pink & moist  • CRT ~2s  • Temp ≥100°F  • Femoral pulses  strong or bounding | • HR: C 60-140  • HR: F 120-200  • Sinus rhythm | • RR regular  • No stridor  • No abdominal  component | • Partial thickness abrasion or laceration  • No ocular fluorescein uptake | • Weight bearing in 3 or 4 limbs  • No palpable fracture  • No joint laxity | • Conscious, alert to slightly dull  Interested in surroundings  • Normal spinal reflexes  • Purposeful movement and nociception in all 4 |
| 1 | • MM hyperemic / pale  • MM tacky  • CRT <2s  • Temp ≥100°F  • Femoral pulses fair | • HR: C 140-180  • HR: F 200-260  • Sinus rhythm or VPCs <20/min | • RR mildly ↑  • Effort mildly ↑  • Some abdominal  component  • URT sounds  mildly ↑ | • Full thickness abrasion or laceration  • No deep tissue involvement  • Corneal laceration or ulcer  • No corneal perforation | • Closed appendicular or rib #  • Any mandibular #  • Single joint laxity or luxation including SI  • Pelvic # with unilateral  intact SI-ileum-acetabulum  • Single limb # at or below carpus/tarsus | • Obtunded  • Abnormal spinal reflexes  • Motor and nociception in all 4 |
| 2 | • MM very pale pink  • MM very tacky  • CRT 2-3s  • Temp <100°F  • Femoral pulses poor | • HR: C >180  • HR: F >260  • Consistent arrhythmia | • RR mildly ↑  • Effort mildly ↑  • Some abdominal  component  • URT sounds  mildly ↑ | • Full thickness wound with deep tissue involvement  • Artery, nerve, muscle all intact  • Corneal perforation or proptosis | • Multiple grade 1 conditions  • Single long bone open # above carpus / tarsus with cortical bone preserved  • Non-mandibular skull # | • Stuporous  • Absent motor in ≥2 limbs  • Deep pain intact in ≥3 limbs  • Decreased tail or anal tone |
| 3 | • MM gray/blue/white  • CRT >3s  • Temp <100°F  • No femoral pulses | • HR: C <60  • HR: F <120  • Erratic rhythm | • Gasping, agonal or irregular respiration  • Effort markedly ↑  • Little or no air movement | • Penetration into abdomen or thorax  • Full thickness wound with deep tissue involvement  • Compromised artery, nerve, muscle | • Vertebral body fracture / luxation (not coccygeal)  • Multiple long bone open # above carpus / tarsus  • Single long bone open # above carpus/tarsus with loss of cortical bone | • Comatose  • Seizures  • Deep pain –ve in ≥2 limbs  • Absent tail or perianal deep pain |

**Modified Glasgow Coma Scale score (MGCS)**

The MCGS uses assessments of motor function, cranial nerves and mentation to assign a score out of 18 to the level of central nervous system dysfunction (Platt et al. 2001). The score is assigned by matching patient evaluations to pre-defined objective statements per the table below. A score of 18 is normal.


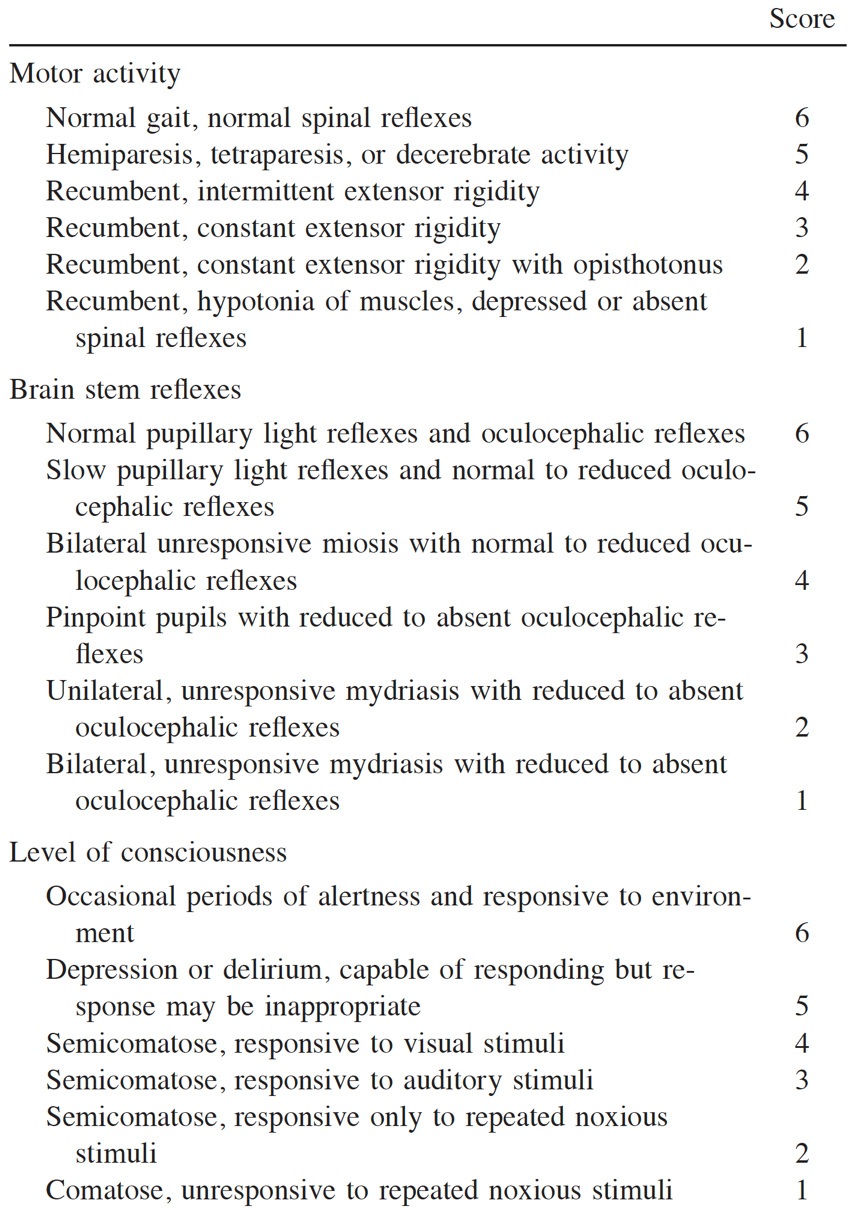


**Sequential Organ Failure Assessment (SOFA)**

The SOFA score was originally reported for assessment of human intensive care patients (Vincent et al. 1996). The SOFA score was modified by Ripanti et al. (2012) for use in critically ill dogs. The score is calculated as the sum of the assigned value (0-4) for each of 6 categories of organ function per the table below.


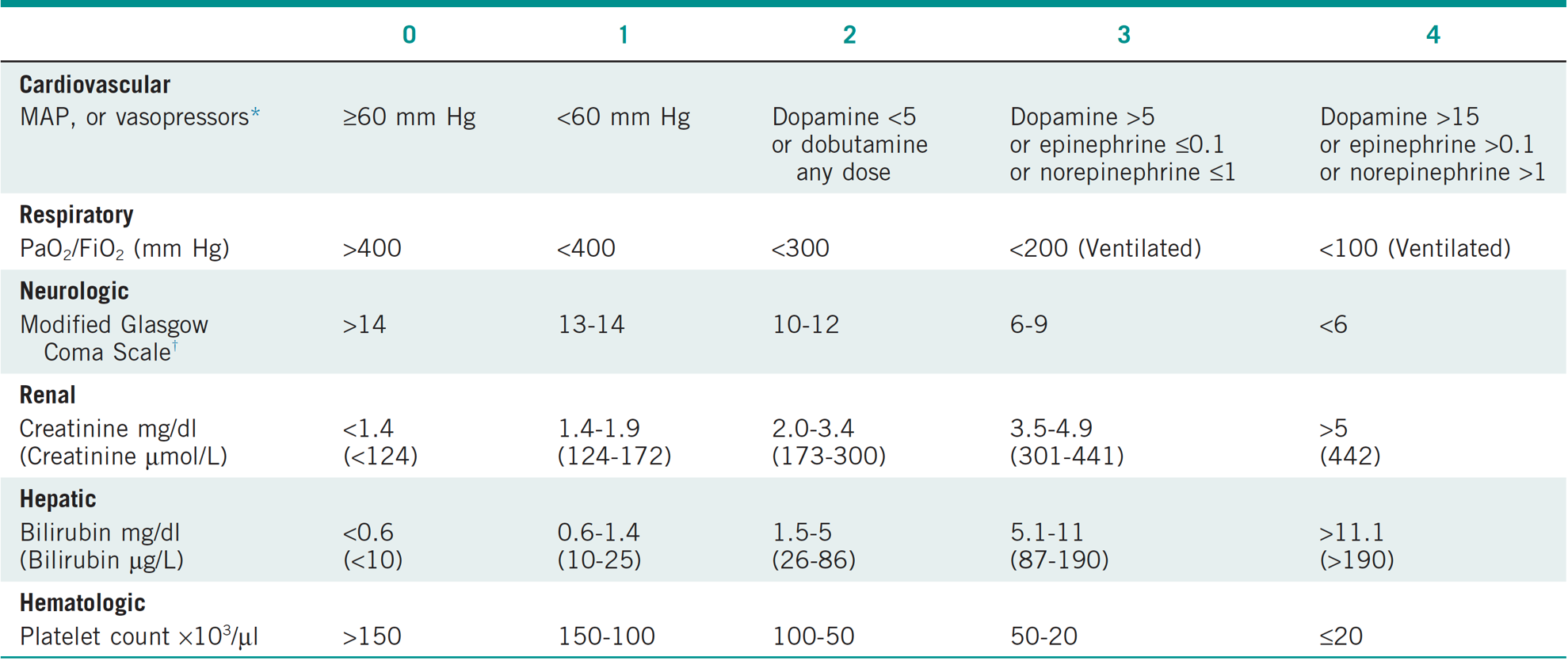


**Shock index**

The shock index (SI) was calculated as heart rate (bpm) divided by systolic arterial blood pressure (mmHg) per Peterson et al. (2013):

SI = HR/SBP

**Survival Prediction Index-2 (SPI2)**

The second survival prediction index was developed by King and others (xxxx) for the assessment of canine patients in the intensive care unit. The score is calculated by inputting the measured parameters into a logistic regression equation as follows:

Logit P = 0.3273 + (0.0108 • MAP) – (0.0102 • respiratory rate) – (0.2183 • creatinine) + (0.0164 • PCV) + (0.3553 • albumin) – (0.1184 • age) – (0.8069 • medical vs surgical status)

Where MAP is mean arterial pressure in mmHG, respiratory rate is measured in breaths per minute, creatinine concentration is measured in mg/dL, PCV is packed cell volume measured in %, albumin concentration is measured in mg/dL, and age is in years. All trauma patients in the current study were coded as medical for the purposes of SPI2 calculation, irrespective of whether they underwent surgery or not.

The percentage probability of survival can then be calculated as:

Probability = 0.3273 + log([1 – 0.635]/0.635) + log(SPI2/[1 – SPI2])

**References**

Hayes G, Mathews K, Doig G, Kruth S, Boston S, Nykamp S, et al. The acute patient physiologic and laboratory evaluation (APPLE) score: a severity of illness stratification system for hospitalized dogs. *J Vet Intern Med* (2010) 24, 1034-47. doi: 10.1111/j.1939-1676.2010.0552.x.

King LG, Wohl JS, Manning AM, Hackner SG, Raffe MR, Maislin G. Evaluation of the survival prediction index as a model of risk stratification for clinical research in dogs admitted to intensive care units at four locations. *Am J Vet Res* (2001) 62, 948-54.

Peterson KL, Hardy BT, Hall K. Assessment of shock index in healthy dogs and dogs in hemorrhagic shock. *J Vet Emerg Crit Care* (2013) 23, 545-50. doi: 10.1111/vec.12090.

Platt SR, Radaelli ST, McDonnell JJ. The prognostic value of the modified Glasgow Coma Scale in head trauma in dogs. *J Vet Intern Med* (2001) 15, 581-4.

Ripanti D, Dino G, Piovano G, Farca A. Application of the Sequential Organ Failure Assessment Score to predict outcome in critically ill dogs: preliminary results. *Schweiz Arch Tierheilkd* (2012) 154, 325-30. doi: 10.1024/0036-7281/a000356.

Rockar RA, Drobatz KS, Shofer FS. Development Of A Scoring System For The Veterinary Trauma Patient. *J Vet Emerg Crit Care* (1994) 4, 77-83. doi: 10.1111/j.1476-4431.1994.tb00118.x.

Vincent JL, Moreno R, Takala J, Willatts S, DeMendonca A, Bruining H, et al. The SOFA (sepsis-related organ failure assessment) score to describe organ dysfunction/failure. *Intensive Care Med* (1996) 22, 707-10. doi: 10.1007/bf01709751.
