# Supplementary material for: High Mobility Group Box-1 and Pro-inflammatory Cytokines Are Increased in Dogs After Trauma but Do Not Predict Survival
Source: Front Vet Sci. 2018 Jul 30;5:179. doi: 10.3389/fvets.2018.00179 (PMC6077187; doi:10.3389/fvets.2018.00179)
Supplement: Supplementary Table 1 — Summary statistics for complete blood count and serum chemistry panels collected at presentation to the study institution. Data are presented in SI units. Institution reference intervals are indicated in parentheses. [file Table_1.DOCX]

Supplementary Table 1. Summary statistics for complete blood count and serum chemistry panels collected at presentation to the study institution. Data are presented in SI units. Institution reference intervals are indicated in parentheses.

| **Parameter** | **n** | **Min** | **25%** | **Median** | **75%** | **Max** | **Mean** | **SD** |
| --- | --- | --- | --- | --- | --- | --- | --- | --- |
| Hematocrit (%) ^*^  (41-58) | 46 | 27 | 40 | 47 | 53.5 | 72 | 46.13 | 9.4 |
| Leukocytes (x10^9^/L)  (5.7-14.2) | 46 | 6.4 | 9.175 | 10.95 | 14.9 | 26.4 | 12.96 | 5.6 |
| Neutrophils (x10^9^/L)  (2.7-9.4) | 46 | 3 | 6.5 | 8.7 | 13.25 | 22.9 | 10.57 | 5.6 |
| Band neutrophils (x10^9^/L)  (0.0-0.1) | 46 | 0 | 0 | 0 | 0.3 | 1.2 | 0.23 | 0.4 |
| Lymphocytes (x10^9^/L)  (0.9-4.7) | 46 | 0.3 | 0.8 | 1.2 | 1.825 | 3.5 | 1.36 | 0.8 |
| Monocytes (x10^9^/L)  (0.1-1.3) | 46 | 0.1 | 0.275 | 0.5 | 0.825 | 2.3 | 0.65 | 0.6 |
| Platelets (x10^9^/L) ^*^  (186-545) | 46 | 60 | 181 | 239 | 300.3 | 487 | 245 | 96 |
| Sodium (mmol/L) ^*^  (143-150) | 46 | 143 | 147 | 148 | 151 | 157 | 149 | 3.2 |
| Potassium (mmol/L)  (4.1-5.4) | 46 | 3.2 | 3.875 | 4.1 | 4.225 | 5.2 | 4.07 | 0.4 |
| Chloride (mmol/L)  (106-114) | 46 | 102 | 107.8 | 109.5 | 114 | 127 | 110.8 | 5 |
| Bicarbonate (mmol/L)  (14-24) | 46 | 7 | 17 | 19.5 | 22 | 25 | 19.07 | 3.5 |
| Anion Gap (mmol/L)  (17-27) | 46 | 15 | 20 | 23.5 | 26 | 40 | 23.15 | 4.5 |
| BUN (mmol/L)  (3.2-9.3) | 46 | 1.4 | 5.1 | 6.8 | 8.2 | 15.7 | 6.8 | 2.5 |
| Creatinine (mmol/L)  (53.0-123.8) | 46 | 26.5 | 53.0 | 70.7 | 88.4 | 176.8 | 76.9 | 35.4 |
| Calcium (mmol/L) ^*^  (2.35-2.78) | 46 | 1.88 | 2.29 | 2.44 | 2.63 | 2.83 | 2.43 | 0.25 |
| Phosphate (mmol/L) ^*^  (0.87-1.74) | 46 | 0.48 | 1.03 | 1.41 | 1.73 | 3.00 | 1.47 | 0.61 |
| Magnesium (mmol/L)  (0.75-1.05) | 46 | 0.60 | 0.75 | 0.85 | 0.95 | 1.55 | 0.88 | 0.20 |
| Total Protein (g/L) ^*^  (55-72) | 46 | 23.0 | 47.3 | 54.0 | 64.0 | 94.0 | 54.8 | 13.0 |
| Albumin (g/L) ^*^  (32-41) | 46 | 15 | 28.8 | 33 | 35.3 | 44 | 31.9 | 6.0 |
| Globulin (g/L) ^*^  (19-37) | 46 | 8 | 16.8 | 19.5 | 27.0 | 38.0 | 21.6 | 7.0 |
| Glucose (mmol/L)  (3.8-5.8) | 46 | 4.7 | 5.8 | 6.5 | 8.4 | 16.4 | 7.4 | 2.4 |
| ALT (U/L)  (17-95) | 46 | 14 | 98.75 | 364.5 | 1129 | 6273 | 899.8 | 1333 |
| AST (U/L)  (18-56) | 46 | 47 | 125.3 | 338 | 1318 | 4306 | 902.3 | 1135 |
| ALP (U/L)  (7-115) | 46 | 17 | 41.75 | 67 | 136.5 | 834 | 113.9 | 137.1 |
| GGT (U/L)  (0-8) | 46 | 3 | 3 | 3 | 6.25 | 18 | 5.3 | 3.7 |
| T. Bilirubin (µmol/L) ^*^  (0.0-3.4) | 46 | 0.0 | 0.0 | 1.7 | 3.4 | 6.8 | 2.1 | 1.7 |
| Cholesterol (mmol/L) ^*^  (3.5-10.2) | 46 | 1.6 | 3.9 | 5.2 | 6.7 | 9.1 | 5.1 | 1.8 |
| CK (U/L)  (64-314) | 46 | 301 | 1398 | 3371 | 7050 | 51217 | 6765 | 9790 |

^*^ These parameters were normally distributed; the remainder were non-parametric. Abbreviations: ALT, alanine aminotransferase; ALP, alkaline phosphatase; AST, aspartate aminotransferase; CK, creatine kinase; GGT, gamma-glutamyl transpeptidase; T. Bilirubin, total bilirubin.
