# Supplementary material for: High Mobility Group Box-1 and Pro-inflammatory Cytokines Are Increased in Dogs After Trauma but Do Not Predict Survival
Source: Front Vet Sci. 2018 Jul 30;5:179. doi: 10.3389/fvets.2018.00179 (PMC6077187; doi:10.3389/fvets.2018.00179)
Supplement: Supplementary Table 2 — Summary of multivariable logistic regression model assessing the association between HMGB-1 concentrations and survival to discharge accounting for prior assessment by a primary care veterinarian. [file Table_2.docx]

| Summary of independent predictive variables for death at discharge | | | | | | | |
| --- | --- | --- | --- | --- | --- | --- | --- |
| Variable | Coefficient  (B) | | SE | P value | Odds Ratio  Exp (B) | 95% CI for OR | |
|  |  |  |  |  |  | Lower | Upper |
| HMGB-1 | -0.020 | | 0.009 | 0.028 | 0.980 | 0.963 | 0.998 |
| Prior rDVM visit | -2.700 | | 1.315 | 0.040 | 0.067 | 0.005 | 0.885 |
| Constant | 3.976 | | 1.354 |  |  |  |  |
|  | | | | | | | |
| AUROC | | 0.849 | | | | | |
| Hosmer-Lemeshow P value | | 0.066 | | | | | |
| Nagelkerke’s R^2^ | | 0.375 | | | | | |
